# Beckett Hospital, Barnsley

**Published:** 1918-04-06

**Authors:** 


### THE EDITOR'S LETTER-BOX.

### Beckett Hospital, Barnsley.

*To the Editor of* The Hospital.

Sir,—May I reply to your paragraph under "Round the Hospitals" on p. 558 of your last week's issue? As the inquiry was well advertised and no one came forward, does not this prove how feeble the anonymous complaints were? With the exception of the few undesirables, the nurses left in the ordinary way. It is only the ignorant, badly bred person who ever objects to the necessary hospital discipline. These the hospital is well rid of and they will never make nurses. The old order of offering no salary or very little was to attract the better-class probationer; now one must move with the times and give more remuneration, otherwise the small civil hospital must suffer. The military are too attractive with their larger salaries. The hours of duty here are less than most hospitals and much has been done this last year for the general improvement of the nursing staff, including systematic instruction and the increase of salaries. In four and a half years three matrons have been engaged. Doesn't this all make for restlessness and dissatisfaction? As a member of the nursing staff for the last fourteeen months I applaud with others the good work our matron has done.—Yours faithfully, F. E.

